# No Pain Relief with the Rubber Hand Illusion

**DOI:** 10.1371/journal.pone.0052400

**Published:** 2012-12-20

**Authors:** Rahul Mohan, Karin B. Jensen, Valeria I. Petkova, Abishikta Dey, Nadia Barnsley, Martin Ingvar, James H. McAuley, G. Lorimer Moseley, Henrik H. Ehrsson

**Affiliations:** 1 Neuroscience Research Australia, Sydney, Australia; 2 Brain, Body, and Self Laboratory, Department of Neuroscience and Department of Clinical Neuroscience, Osher Center for Integrative Medicine, Stockholm Brain Institute, Karolinska Institutet, Stockholm, Sweden; 3 Sansom Institute for Health Research, University of South Australia, Adelaide, Australia; University of Bologna, Italy

## Abstract

The sense of body ownership can be easily disrupted during illusions and the most common illusion is the rubber hand illusion. An idea that is rapidly gaining popularity in clinical pain medicine is that body ownership illusions can be used to modify pathological pain sensations and induce analgesia. However, this idea has not been empirically evaluated. Two separate research laboratories undertook independent randomized repeated measures experiments, both designed to detect an effect of the rubber hand illusion on experimentally induced hand pain. In Experiment 1, 16 healthy volunteers rated the pain evoked by noxious heat stimuli (5 s duration; interstimulus interval 25 s) of set temperatures (47°, 48° and 49°C) during the rubber hand illusion or during a control condition. There was a main effect of stimulus temperature on pain ratings, but no main effect of condition (p = 0.32), nor a condition x temperature interaction (p = 0.31). In Experiment 2, 20 healthy volunteers underwent quantitative sensory testing to determine heat and cold pain thresholds during the rubber hand illusion or during a control condition. Secondary analyses involved heat and cold detection thresholds and paradoxical heat sensations. Again, there was no main effect of condition on heat pain threshold (p = 0.17), nor on cold pain threshold (p = 0.65), nor on any of the secondary measures (p<0.56 for all). We conclude that the rubber hand illusion does not induce analgesia.

## Introduction

We humans have a continuing experience that we are owners of our bodies, so that when we look at our hands, for example, we immediately recognize them as our own. We can also discriminate between our own pain and that of another. Both body ownership and pain can be easily studied experimentally. For example, scientists manipulate body ownership using the out of body illusion [1], the body-swapping illusions [2], the fake finger illusion [3], the disappearing hand illusion [4] and the swollen hand illusion [5] and the most common approach to manipulate body ownership is the rubber hand illusion [6].

The rubber hand illusion exploits the brain’s predilection for congruent multisensory input such that, by synchronously stroking a rubber hand held in view and the real hand, held out of view, the participant quickly gains the sense of feeling the touch on the rubber hand. The somatosensory stimuli delivered on the rubber hand are mapped according to a visually-based, external frame of reference centered on the arm [7], [8]. The stimuli delivered on the real hand and not seen by the participant, are first mapped according to a somatotopic frame of reference, and then automatically re-mapped into the common external frame of reference [9], [10] such that the seen and felt stimuli are perceptually fused to be one and the same [7].

Recent attention has turned to the consequences of the rubber hand illusion for the real hand - the one that is ‘replaced’. The rubber hand illusion leads to a limb-specific drop in temperature of the real hand, a shift in tactile processing such that tactile information from the real hand are given less weighting by the brain than identical stimuli from the opposite hand [11], and increased reactivity to intradermal histamine [12]. These findings, combined with the observation that threatening the rubber hand evokes protective physiological responses as though one’s own hand had been threatened [13], raises the possibility that the cortical representation of the rubber hand does indeed ‘replace’ that of the real hand. The large amount of research in this area has led to a notion that is gaining popularity in the clinical field – that bodily illusions such as the rubber hand illusion can be used to relieve pain. This popularity may reflect extrapolation from the apparent success of other treatments that target the brain, for example graded motor imagery [14], [15], [16], [17] (see [18] for review), but the enthusiasm with which the extrapolation has been endorsed is remarkable considering the lack of supportive evidence. To our knowledge, the only empirical data relating to body ownership and pain is the finding of a higher pressure pain threshold on the index finger during a version of the full-body illusion protocol [19] than during control conditions [20].

The current experiments aimed to detect a clinically useful analgesic effect of the rubber hand illusion. The experiments were undertaken by two independent research teams in different laboratories. Both experiments involved the use of thermal stimuli. Both experiments were designed to test the hypothesis that the rubber hand illusion would have an analgesic effect on the pain evoked by noxious stimuli delivered on the real arm.

## Methods – Experiment 1

### Participants

Sample size was based on established variability data from experimentally induced heat-pain experiments. To detect a clinically-relevant effect of 20 points on 100 point visual analogue scale [21] with 80% power at α = 0.05, using a repeated measures within-subjects design, we required 15 participants. We recruited 16 participants (8 females, mean age 25, SD = 7). All participants were naïve to the purposes of the experiment. The Ethical Review Board of Karolinska Institutet approved the experimental protocol and was conducted in accordance with the Declaration of Helsinki. Written informed consent was obtained from each participant.

### Design

Randomised repeated measures experiment.

### Procedure

#### Noxious stimulus

Pain was induced via noxious stimulation to the dorsum of the hand using a 30 mm×30 mm heat probe from the Pathway ATS thermal pain system, Medoc Israel (http://www.medoc-web.com). The heat probe was attached to the hand using an elastic strap, allowing the probe to rest on the hand with light pressure to the skin. Pathway ATS thermal pain system, Medoc Israel (http://www.medoc-web.com), which was driven by TSA-2001 software via a laptop.

Each subject was calibrated for subjective pain ratings by receiving a series of thermal stimuli with the temperatures 47°C, 48°C and 49°C, given five times each in a randomized order. The duration of each stimulus was 5 seconds, delivered at 30 seconds intervals. At the end of each stimulus, subjects were instructed to rate the pain intensity by putting a mark on a 100 mm horizontal visual analogue scale (VAS) anchored with “no pain” and “worst imaginable pain”. Since a fixed range of temperatures were used for calibration, subjects were instructed to directly inform the experimenter if any of the temperatures were perceived as unbearable, and if this occurred that particular stimulus temperature would instantaneously be omitted.

The mean VAS rating was calculated for each of the three temperatures used during the calibration. The temperatures that best represented each individual’s high pain (60 mm VAS) were determined and used in the two subsequent rubber hand illusion pain runs. From this individual high pain we subtracted 1.5°C to obtain the individual low pain temperature.

#### Rubber hand illusion

**Figure 1:**
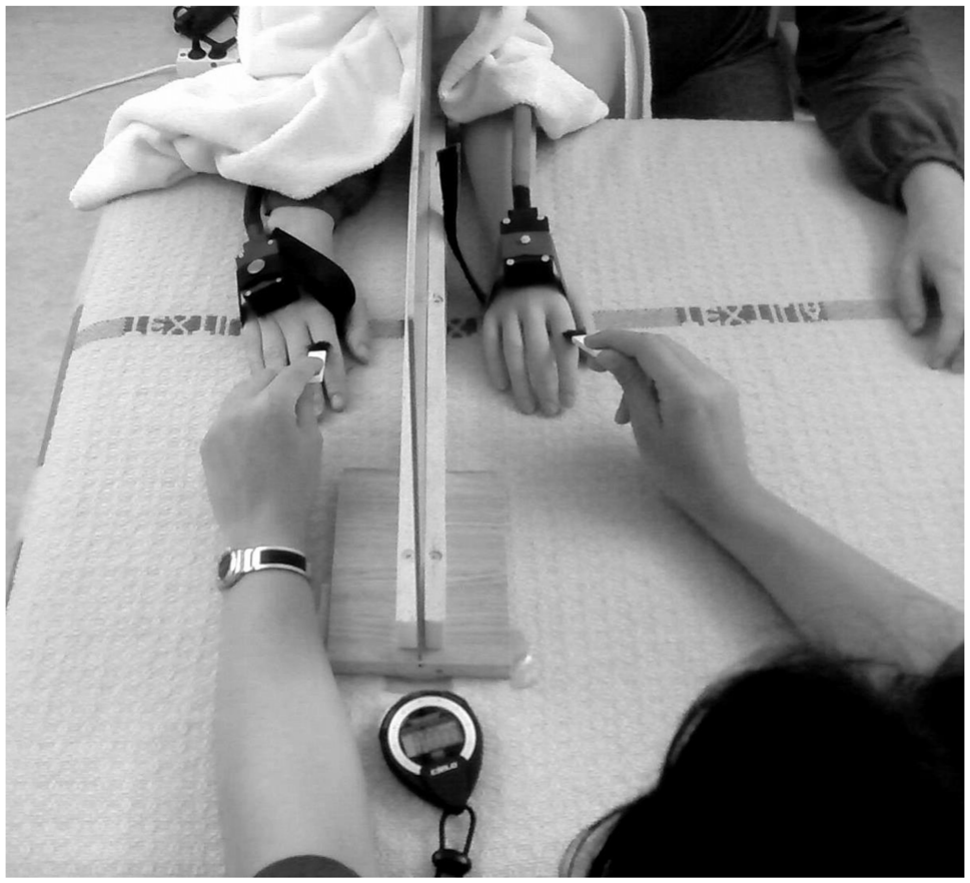
Experimental 1 & 2– experimental set-up.

The participants were seated with their arms resting prone on a table (Fig. 1). A life-size right cosmetic hand prosthesis was placed on the table twenty centimeters to the right of the midline of the participants’ body. The real right hand was hidden behind a screen at a distance of 15 centimeters from the rubber hand. Two identical heat probes were attached to both the rubber hand and the real right hand. A towel was placed over the proximal ends of the arms to cover the gap between the rubber arm and the person’s body. All participants were instructed to look at the rubber hand. The left hand was placed in full view twenty centimeters to the left of the midline of the body. The participants were holding a pen with their left hand and were instructed to use it to mark the pain ratings on the visual analogue scale described above.

Two identical brushes were used to stroke the right real and the right rubber hands either synchronously (corresponding to the illusion condition) or asynchronously (providing the control condition). The touches were delivered to the corresponding parts of the index and middle fingers of the right rubber hand and right real hand. The dorsal side of the hand was not stroked as the head probe was placed there. An irregular, but synchronous, rhythm of brushing was chosen to enhance the illusion since this mode of stimulation is known to maximize the traditional rubber hand illusion (Petkova & Ehrsson, 2009). The brushing in the asynchronous condition was in an irregular and alternating pattern. The participants were explicitly instructed not to move their right hand behind the occluding screen. Each session of synchronous or asynchronous brushing was 1.5 minutes long.

The heat stimulus was applied at the end of each session while the brushing was still ongoing. After the heat stimulation the participant was requested to mark the pain ratings. The next session of brushing began immediately after that subjects have performed this pain rating. The experiment consisted of a total of 16 sessions. The calibration runs were performed on the dorsum of the left hand and the experimental runs were performed on the dorsum of the right hand. In addition after the first 8 experimental sessions the location of the heat probe was changed again. This was done in order to prevent increased sensitivity to the pain stimulus over time. The order of the two temperature stimuli as well as the order of the synch/async runs was counterbalanced in the following way:

SH AL AH SH SL AH AL SL break SL AH AL SL SH AL AH SH (4 participants).

AL SH SL AL AH SL SH AH break AH SL SH AH AL SH SL AL (4 participants).

SL AH AL SL SH AL AH SH break SH AL AH SH SL AH AL SL (4 participants).

AH SL SH AH AL SH SL AL break AL SH SL AL AH SL SH AH (4 participants).

where S stands for synchronous brushing (illusion); A stands for asynchronous brushing (control); H stands for high temperature and L stands for low temperature.

**Figure 2:**
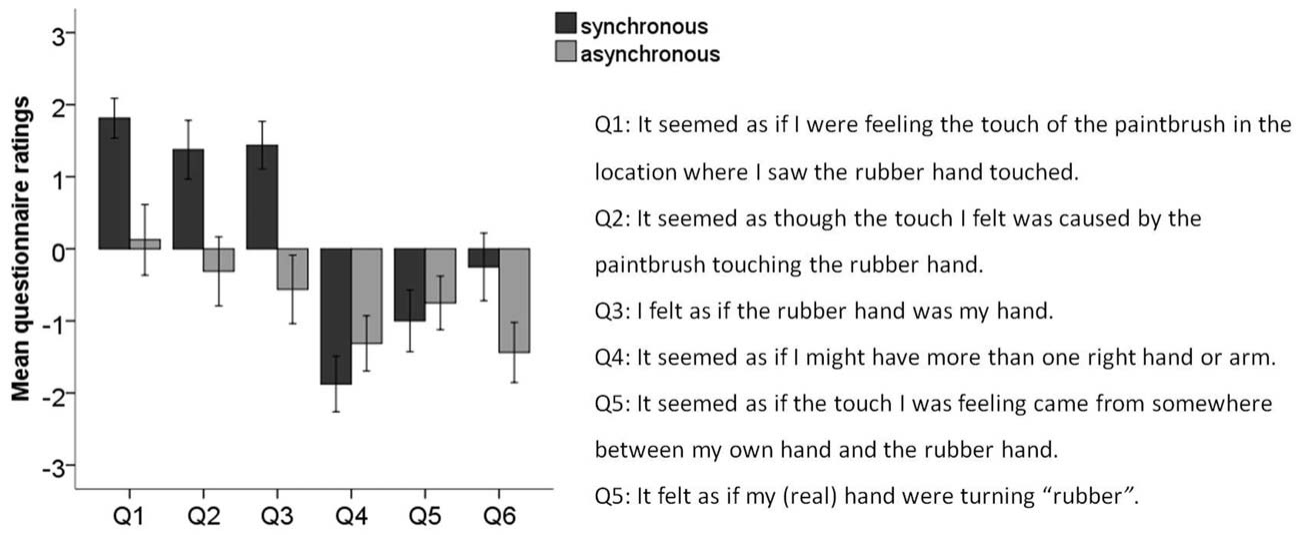
Experiment 1 results: Mean (columns) and standard error (error bars) for ratings to the illusion questions (Q1–Q3) and control questions (Q4–Q6) for the illusion (dark gray) and the control (light gray) conditions. The scale ranges from ‘+3′ (‘I agree strongly’) to ‘−3′ (‘I disagree strongly’) with ‘0′ denoting uncertainty.

At the end of the experiment we had two sessions of synchronous and asynchronous brushing of 1.5 minute each to confirm successful induction of the rubber hand illusion. Half of the participants started with the synchronous condition and the other half started with the asynchronous condition. At the end of each session, the participants were asked to fill out a short version of the questionnaire used in the original rubber hand illusion study [6], which consisted of six statements about the experiences they might have had during the stimulation. Three statements (Q1–Q3) were designed to capture different aspects of the illusory perception related to the sensation of touches on the rubber hand and the feeling of ownership of that hand. Statements Q4–Q6 served as control questions for task compliance and susceptibility effects (Figure 2). The participants were asked to rate their level of agreement with the statements on a seven-point Likert scale with a range from “+3” (agree very strongly) to “−3” (disagree very strongly) where “0″ corresponded to neither agreeing nor disagreeing. We administered thw rubber hand illusion questionnaire after the pain experiment to ensure that the participant where genuinely naïve about the illusion when they reported the perceived pain.

### Analyses

This experiment involved a 2×2 full factorial ANOVA. The first factor was temperature (high or low) and the second factor was condition (rubber hand illusion or control). Significance was set at α = 0.05.

## Results

### Rubber Hand Illusion

Fifteen out of the sixteen participants felt as though the rubber hand was their real hand (ratings on statement Q1 of ≥ +1) when it was brushed in synchrony with their right hand. The rating scores were significantly greater on the illusion questions than on the control questions, and this effect was significantly greater after a period of synchronous stimulation. There was a main effect of “Condition” (synchronous, asynchronous) (F(1, 15) = 9.784, p = .007) and a main effect of “Question type” (illusion, control) (F(1, 15) = 35.951, p<.001), and, crucially, a significant interaction between the two factors (F(1, 15) = 27.778, p<.001) (Figure 2).

### Pain

**Figure 3:**
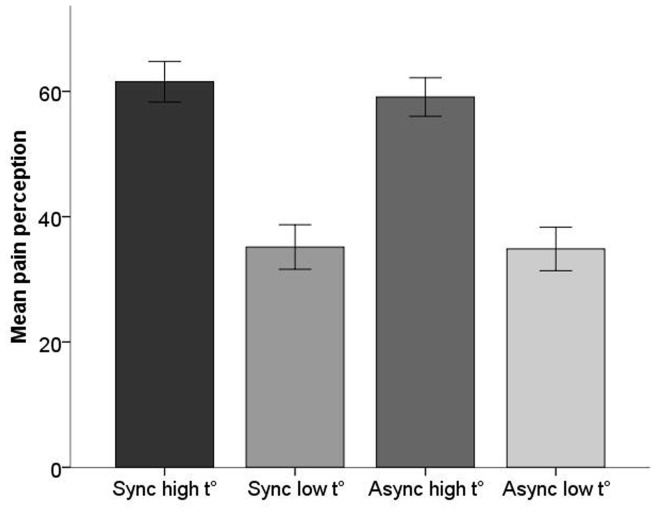
Experiment 1 results: Mean (columns) and standard error (error bars) for pain ratings after exposure to synchronous versus asynchronous visuo-tactile stimulation with the rubber hand for high (painful) and low (non-painful) temperature stimulation.

The Rubber Hand Illusion did not modulate the experience of the low and the high temperature stimuli. In a 2×2 ANOVA, we found a significant main effect of Temperature (low, high) (F(1,15) = 64.400, p<.001), but no main effect of “Condition” (synchronous, asynchronous) (F(1,15) = 1.056, p = .320), and no significant interaction (F(1,15) = 1.101, p = .311) (Figure 3). Finally, we found no significant correlation between the strength of the rated illusory ownership and the pain intensity ratings. Thus, we found no evidence in our data that ownership modulates perceived pain intensity.

## Methods - Experiment 2

### Participants

Because this study was investigating pain thresholds, the clinically relevant effect should be smaller [22]. We aimed to recruit 20 healthy participants, which would give us 80% power to detect a moderate effect with α = 0.05. Participants were naïve to the purposes of the experiment. We recruited through on-line advertisements on Facebook, our laboratory blog at bodyinmind.org, and Twitter. Eligibility criteria were age 18–80 years old, English speaking and capable of giving informed consent. Exclusion criteria were existing pain in any part of the body, existing or diagnosed psychiatric or neurological illness, recent (past 24 hours) use of anti-inflammatory drugs, pregnancy, blindness or any medical condition that affected their arm. The study was approved by the University of New South Wales Ethics Committee, and was conducted in accordance with the Declaration of Helsinki. Written informed consent was obtained from each participant.

### Design

Randomised repeated measures experiment.

### Procedure

#### Noxious stimulus

We undertook an established Quantitative Sensory Testing (QST) protocol recommended by the German Research Network on Neuropathic Pain (DFNS)(CITE). This experiment also used a Pathway ATS thermal pain system, Medoc Israel (http://www.medoc-web.com), which was driven by TSA-2001 software via a laptop. The 30 mm×30 mm probe was attached to the dorsal surface of the experimental hand and a fake probe was attached to the rubber hand. The following QST components were tested: thermal detection thresholds for perception of cold (CDT), warm (WDT) and thermal sensory limen (TSL), thermal pain thresholds for cold (CPT) and hot (HPT) stimuli.

As per the established protocol, the order of measures was consistent across participants (Table 1). For each measure, the participant responded by pressing a button with the hand that was not being used for the experiment (see below). The button stopped the delivery of stimuli which was ramped (1°C/s) for all types of stimuli. The baseline temperature was 32°C and the return rate was 1°C/s for thermal detection thresholds and 5°C/s for pain detection thresholds. The cut off temperatures were 0°C and 50°C. The mean threshold temperature for four consecutive stimuli was used for analysis.

**Table 1:** Raw data for experiment 2.

Measure	Mean	Std Deviation	T score	Significance
CDT	−0.07	1.0505	0.298	0.769
WDT	−0.1040	1.5104	−.308	0.761
TSL	−0.220	1.6571	−0.594	0.560
CPT	−0.7415	7.1403	−0.464	0.648
HPT	−0.9515	2.9575	−1.439	0.166

CDT = old detection threshold; WDT = warm detection threshold; TSL = thermal sensory limen; CPT = cold pain threshold; HPT = heat pain threshold. Mean and standard (std) deviation shown in degrees Celsius.

#### Rubber hand illusion

The study was conducted in an air-conditioned room with no distractions or interruptions for the duration of the experiment. The participants were given 10–15 minutes to acclimatise to the room before commencing the experiment.

A random numbers table was used to place each recruited participant into either the experimental condition or the control condition. The use of a left or a right rubber hand was also randomised using a random numbers table. The procotol for inducing the rubber hand illusion was very similar to that used in Experiment 1, with the exception of evaluation. Participants completed the questionnaire for each condition after the experiment was completed. Participants also completed a 0–10 numerical rating scale to rate the vividness of the illusion:

“On a scale of 1–10 how strong was the illusion that the rubber hand was your own hand? 1 being not at all and 10 being a very strong illusion”.

Once the illusion was in place, thermal testing was commenced. If a participant did not experience the illusion, the experiment was terminated. For those who did report a vivid illusion, the same stroking technique was used for 20 seconds to reinforce the illusion, at three predetermined intervals; before the TSL, CPT and HPT tests. The illusion was assessed after each test.

### Analyses

Paired t-tests were used to compare the primary outcomes, CPT and HPT, between conditions. WDT, CDT, TSL, HPT, and CPT were compared using paired T-tests. We did not correct for multiple measures because we wanted to minimise the likelihood of a type II error. That is we wanted to minimize the risk of missing an effect of the rubber hand illusion on pain rather than falsely detecting one. To verify that the illusion was effective, we undertook a 2 (condition: RHI or control) ×2 (question type: illusion or control) ANOVA on questionnaire data. We also undertook a 2 (condition: RHI or control) ×4 (test occasion: 1–4) ANOVA on participants’ responses to the numerical rating scale for illusion vividness.

## Results

### Rubber Hand Illusion

Twelve participants were randomly allocated to the rubber hand illusion condition first and eight to the control condition first. Fifteen participants were randomly assigned to use the right hand for the experiment and five were randomly assigned to use the left hand for the experiment. The participant’s handedness was not taken into consideration, although two of the participants reported to be left-handed and one reported to be ambidextrous.

The results of inducing the illusion were almost identical to those of Experiment 1. The rating scores were significantly greater on the illusion questions than they were on the control questions. There was a main effect of “Condition” (synchronous, asynchronous) (F(1, 19) = 96.13, p<0.001) and a main effect of “Question” (F(1, 19) = 123.74; p<0.001) and, crucially, a significant interaction between the two factors (F(1, 19) = 88.20, p<0.001). The ANOVA of numerical rating scale data corroborated the questionnaire data. There was a main effect of “Condition” (F(1,19) = 72.93; p<0.001) but no effect of “Test occasion” (p = 0.18) and no Condition×Test occasion interaction (p = 0.30).

**Figure 4:**
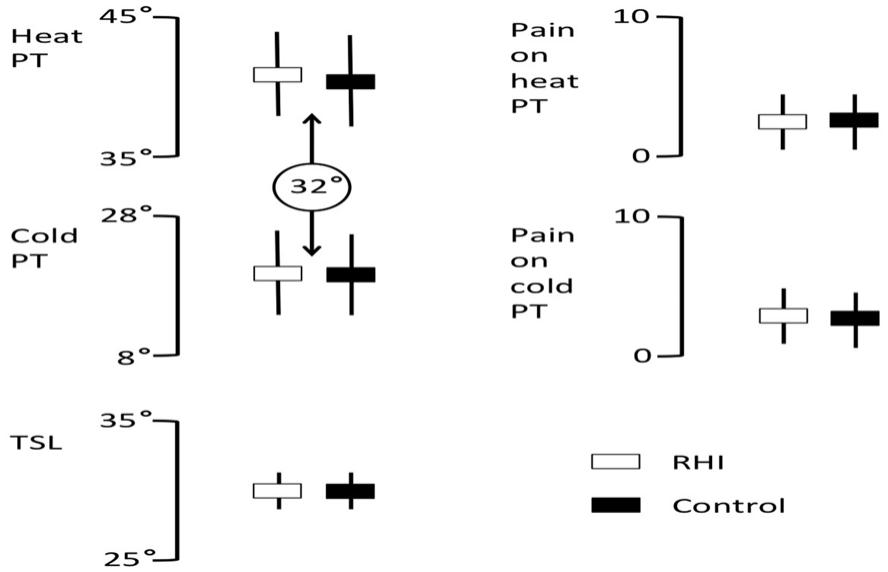
Experiment 2 results: Mean (rectangle) and standard deviation (error bars) heat pain threshold (HPT), cold pain thresholds (CPT) and thermal sensory lymen (TSL) in °C for the rubber hand illusion (rubber hand illusion; open shapes) control conditions. Right panels show pain ratings on a 0–10 scale for the pain experienced for the threshold stimuli for heat pain threshold and cold pain threshold condition. The circled 32° reflects the baseline from which a ramping stimulus was used to determine both heat PT and cold PT.

There was no effect of the rubber hand illusion on CPT (t = −4.64, p = 0.648) or HPT (t = −1.439, p = 0.166). Similarly, there was no effect of the rubber hand illusion on WDT (t = −0.308, p = .761), CDT (t = −0.298, = 0.769) or TSL (t = −0.594, p = 0.560) (Fig. 4).

## Discussion

We hypothesised that the rubber hand illusion would have an analgesic effect for stimuli delivered to the real arm. In two experiments, undertaken by independent research teams and both powered to detect a clinically relevant effect, we failed to detect any modulation of pain thresholds, nor of pain evoked by individually calibrated high and low painful stimuli, in association with the rubber hand illusion.

One might predict that a multisensory illusion that brings into conflict different spatial frames of reference that disrupt thermoregulation [11] and immunoregulation (histamine reactivity) [12] might also disrupt nociceptive processing and thereby reduce pain. This prediction is supported by a recent study that found that crossing the arms reduced the intensity of pain in response to noxious stimulation of a hand [23]. That study also showed that crossing the arms had no effect on early stages of noxious processing, but it did have an effect on the later stages of processing, consistent with disruption of the spatial transformation of the stimulus and the production of a perception of the event. However, the present experiments do not add support to the prediction of analgesia via conflicted frames of reference because although the rubber hand illusion clearly disrupts spatial transformation of the stimulus and perception (see [24] for review), it does not reduce pain. The crossed hands analgesic effect involves a much stronger side-to-side mismatch between somatotopic and spatial frames of reference than the rubber hand illusion does, so it remains possible that modifying the rubber hand illusion specifically to maximize the conflict, for example, by adjusting the alignment or orientation of the rubber hand sufficiently to disrupt spatial processing but not to disrupt the vividness of the illusion, might still reduce pain. Clearly further experiments are required.

The present results are intriguing because they indicate that it is possible to attribute pain to an artificial limb and that the intensity of pain is unchanged, just as the intensity of touch may be unchanged in response to tactile non-noxious stimuli [25]. We can infer that the pain was felt in the rubber hand because if it was felt in the real hand, the illusion would probably be broken. In the present two experiments, the heat probes on the rubber hand might have facilitated the “visual capture of pain” as suggested by pilot experiments. These observations together with the study by Capelari and colleagues [26], (as well as unpublished pilots experiments carried out independently by GLM and HHE), suggest that pain can be referred to external objects (e.g. rubber hands) as long as these objects are being perceived as part of one’s own body, that is, they are incorporated into the multisensory body representation. This view is consistent with previous reports that the rubber hand illusion involves sufficient embodiment of the rubber hand so as to evoke regular protective responses in response to threatening stimuli [13], but extends it by demonstrating that the localisation of pain during the rubber hand illusion involves similar mechanisms to those for the localisation of touch. This is important because it is commonly assumed that pain is not referred to external objects, which is in contrast to touch - touch can be referred to the tips of tools for example [27]. Hence, one implication of our results is that the various components of pain can be manipulated independently. That is, the location of pain can be fundamentally manipulated such that it is felt at an external object that is known by the participant to be insensate, without modulating the intensity or unpleasantness of the pain, as indicated by pain report. The ability to experience pain is clearly important for survival and the hardwired nature of nociceptive pathways supports the critical role of pain in protecting the integrity of our tissues. The motivational component of pain however, is unaffected even when localisation of the perceived threat is erroneous, as it is in the rubber hand illusion, a finding that supports previous work by Ehrsson et al (2007) [8], in which the bodily response to threat is maintained even if our body representation is manipulated.

Although the present experiments suggest that the intensity of thermal pain responses in healthy individuals is not modulated by the illusory changes in limb ownership, it is not clear whether the rubber hand illusion might modulate centrally mediated chronic pain. There is growing evidence that a wider treatment paradigm that incorporates mirror therapy, graded motor imagery, reduces pain and disability in both phantom limb pain and complex regional pain syndrome [22]–[24]; [7]. That such treatments seem to offer benefits suggests that investigation of the utility of the rubber hand illusion for central or pathological pain is still warranted even in the light of the present negative results with respect to nociceptive pain in healthy individuals.

The observation that pain intensity was not modulated by a limb ownership illusion, also begs the question if full-body illusions would be more effective in this respect (see [20]). During the full-body version of the rubber hand illusion, changes in ownership are elicited for all body parts simultaneously when viewing a mannequin from the first person perspective in near-personal space [2], [28], during the “out-of-body illusion” the sense of self is displaced several meters from the seen real body which is “disowned” during congruent visuo-tactile stimulation from the perspective of the cameras at the illusory self location [1], [29], and finally, in Blanke’s full-body illusion set-up the participant is observing oneself (or a mannequin) from the third person perspective in far extrapersonal space and the visual stimuli are delivered to the back of this seen body and the real body [19]. The crucial point for the present discussion is that all these types of full-body illusions involve more extensive changes of the own-body representation and/or greater multisensory conflicts. Thus, future studies should further investigate if these approaches could be used as to modulate nociceptive and central pain. Moreover, if full body illusions can modulate pain by virtue of the greater spatial conflict or whole body disembodiment, they might prove to have utility in reducing the pain of, for example, burn victims.

In conclusion, we contend that the rubber hand illusion does not relieve experimentally induced pain in healthy volunteers. This finding suggests against the utility of the rubber hand illusion as a therapeutic tool for pain relief in a clinical setting, but leaves open the possibility that illusions involving multisensory representation or spatial conflict may be helpful for central or widespread pain.
